# Dyspnea, effort and muscle pain during exercise in lung transplant recipients: an analysis of their association with cardiopulmonary function parameters using machine learning

**DOI:** 10.1186/s12931-020-01535-5

**Published:** 2020-10-15

**Authors:** Fausto Braccioni, Daniele Bottigliengo, Andrea Ermolao, Marco Schiavon, Monica Loy, Maria Rita Marchi, Dario Gregori, Federico Rea, Andrea Vianello

**Affiliations:** 1 Division of Respiratory Pathophysiology, Department of Cardio-Thoracic, Vascular and Public Health Sciences, University Hospital of Padova, Padova, Italy; 2 Division of Biostatistics, Epidemiology and Public Health, Department of Cardio-Thoracic, Vascular and Public Health Sciences, University Hospital of Padova, Padova, Italy; 3 Division of Sport and Exercise Medicine, Department of Medicine, University Hospital of Padova, Padova, Italy; 4 Division of Thoracic Surgery, Department of Cardio-Thoracic, Vascular and Public Health Sciences, University Hospital of Padova, Padova, Italy

**Keywords:** Lung transplantation, Cardiopulmonary function, Exercise testing, Arterial blood gases, Borg scale, Muscle pain

## Abstract

**Background:**

Despite improvement in lung function, most lung transplant (LTx) recipients show an unexpectedly reduced exercise capacity that could be explained by persisting peripheral muscle dysfunction of multifactorial origin. We analyzed the course of symptoms, including dyspnea, muscle effort and muscle pain and its relation with cardiac and pulmonary function parameters during an incremental exercise testing.

**Methods:**

Twenty-four bilateral LTx recipients were evaluated in an observational cross-sectional study. Recruited patients underwent incremental cardio-pulmonary exercise testing (CPET). Arterial blood gases at rest and peak exercise were measured. Dyspnea, muscle effort and muscle pain were scored according to the Borg modified scale. Potential associations between the severity of symptoms and exercise testing parameters were analyzed using a Forest-Tree Machine Learning approach, which accomplishes for a ratio between number of observations and number of screened variables less than unit.

**Results:**

Dyspnea score was significantly associated with maximum power output (WR, watts), and minute ventilation (VE, L/min) at peak exercise. In a controlled subgroup analysis, dyspnea score was a limiting symptom only in LTx recipients who reached the higher levels of WR (≥ 101 watts) and V_E_ (≥ 53 L/min). Muscle effort score was significantly associated with breathing reserve as percent of maximal voluntary ventilation (BR%MVV). The lower the BR%MVV at peak exercise (< 32) the higher the muscle effort perception. Muscle pain score was significantly associated with VO_2_ peak, arterial [HCO_3_^−^] at rest, and V_E_/VCO_2_ slope. In a subgroup analysis, muscle pain was the limiting symptom in LTx recipients with a lower VO_2_ peak (< 15 mL/Kg/min) and a higher V_E_/VCO_2_ slope (≥ 32).

**Conclusions:**

The majority of our LTx recipients reported peripheral limitation as the prevalent reason for exercise termination. Muscle pain at peak exercise was strictly associated with basal and exercise-induced metabolic altered pathways. The onset of dyspnea (breathing effort) was associated with the intensity of ventilatory response to meet metabolic demands for increasing WR. Our study suggests that only an accurate assessment of symptoms combined with cardio-pulmonary parameters allows a correct interpretation of exercise limitation and a tailored exercise prescription. The role and mechanisms of muscle pain during exercise in LTx recipients requires further investigations.

## Background

Despite the improvement in lung function, most lung transplant (LTx) recipients show an unexpectedly reduced exercise capacity that could be explained by persisting peripheral muscle dysfunction of multifactorial origin [1]. In the absence of complications, LTx recipients maintain a satisfactory cardiorespiratory function and are able to sustain the levels of physical activity required for daily life [2]. However, when LTx recipients are evaluated by incremental cardiopulmonary exercise testing (CPET), they may show a reduction in the maximal workloads (WR) and oxygen uptake (VO_2_ peak), which usually do not exceed 40–60% of the predicted values. Among the main factors limiting maximum exercise capacity, reduced oxygen extraction related to skeletal muscle dysfunction largely predominates any other limitation. Indeed, cardiac and ventilatory responses are typically normal in LTx recipients. As a result, one of the most common symptoms experienced in lung transplant population is muscle fatigue, impacting on the physical dimension of quality of life [3]. During incremental exercise, many sensations may be discriminated and reproducibly scaled, the most useful being those that represent the combination of several physiologic mechanisms and are experienced as a sense of effort arising in the active muscles, and/or an effort of breathing, and/or pain. Unlike other sensations, the measurement of muscle pain intensity during exercise is difficult because verbal description cannot be simply related to a scale that spans from zero to maximum [4]. However, some authors showed that during incremental cycling exercise, the measurement of perceived pain using the Borg scale increases as a power function of work intensity and this behavior is similar to perceived effort [5]. The possibility to detect symptoms limiting incremental exercise testing, in particular muscle effort and muscle pain, is critical in order to optimize treatment and rehabilitation program for LTx recipients.

The current study is aimed to analyze the role of cardiopulmonary exercise testing parameters on symptoms, including dyspnea, muscle effort, and muscle pain, in view of more tailored exercise prescription in uncomplicated LTx recipients.

## Methods

This observational cross-sectional study was conducted in a tertiary teaching Hospital located in Northeast Italy. The study was approved by the Ethics Committee for Clinical Trials of the Province of Padova (Veneto Region, Italy). All patients provided written informed consent to participate in the study.

### Subjects

Between March 2018 and May 2019, we prospectively enrolled twenty-four bilateral LTx recipients. Reasons for LTx were as follows: cystic fibrosis (13 patients), idiopathic pulmonary fibrosis (6 patients), chronic obstructive pulmonary disease (2 patients), lymphangioleiomyomatosis (1 patient), graft versus host disease (1 patient), Langerhans cell histiocytosis (1 patient). All LTx recipients received immunosuppressive drug treatment including association of prednisone, cyclosporine, and mycophenolate mofetil. LTx recipients were referred to our center by the Thoracic Surgery Unit of our Hospital for a follow-up visit. At our Center, follow-up evaluation includes pulmonary function testing (PFTs), blood gas analysis (ABGs), six-minute walking test (6MWT), and physical examination. Patients able to sustain incremental exercise testing also perform CPET as an alternative to 6MWT to optimize rehabilitation program. Subjects included in the study were clinically stable and did not show acute complications, exacerbated comorbidities or chronic rejection; all individuals underwent a home-based rehabilitation program.

### Interventions

All recruited subjects underwent an incremental CPET by an electronically braked cycle ergometer (Ergoselect 200, Ergoline GmbH, Bitz, Germany) until exhaustion. CPET parameters were computed according to breath-by-breath analysis and data were displayed using an online computer (Vmax 229 Series Encore, Carefusion, Yorba Linda, CA). Continuous 12-lead telemetry was monitored by a CardioSoft electrocardiogram software (CardioSoft, GE Medical Systems, Milwaukee, WI). All the CPET procedures and pulmonary function tests (PFTs) were performed according to standard guidelines [6, 7]. Chronic rejection score was evaluated following 2001 diagnostic criteria [8]. Arterial blood gases (ABGs) at rest and peak exercise were also measured (Rapidpoint 405, Siemens AG, Munich, Germany). Dyspnea, muscle effort, and muscle pain were scored according to the Borg modified scale at rest and peak exercise.

### Statistical analysis

Continuous variables are represented as median (I and III quartiles); categorical variables are represented as frequencies (percentages). The low number of subjects enrolled in our study poses some issues in the development of the statistical analysis plan. In order to overcome these issues, we carried out statistical analysis using an approach based on random forest (RF), one of the most popular machine learning techniques (MLTs) [9]. RF belongs to the ensemble-of-trees methods, a family of algorithms that make predictions aggregating different regression and classification trees (CART), an algorithm that divides the space of the explanatory variables into different regions by recursive binary splitting and performs prediction in each region [10]. RF works by growing one CART on several bootstrap replicates of the original data and then averaging the predictions returned by each tree. Each tree is built considering only a subset of the available predictors, thus allowing the implementation of several CARTs that can be very different from each other. The main idea is to use several “weak” CARTs that can have high predictive performances when pooled together. The adoption of RF in the current analysis was indeed motivated by the fact that such recursive partitioning on the variable space allows naturally dealing with clinical databases with more variables than subjects [11] which is normally intractable with standard statistical regression models. To identify the exercise testing parameters that were associated with the severity of symptoms, we used the Boruta algorithm, a variable selection approach based on RF [12]. Boruta algorithm aims at identifying which are the relevant predictors that impact the outcome of interest. It basically consists of implementing an RF on an augmented set of covariates, where the additional covariates, called shadow variables, are copies of the original ones obtained by permuting the observations and thus removing the eventual association with the outcome. For each explanatory variable, an importance measure is computed, i.e. the Z-score, which is the average improvement in the predictive performance of the RF with the considered explanatory variable divided by its standard deviation. The important predictors are those that show a Z-score higher than the one observed for the variable with the maximum Z-score among the shadow variables. The procedure is repeated until an importance measure is assigned to each predictor or until the maximum number of RF is reached. A Boruta algorithm was implemented for each of the three symptoms: dyspnea, muscle effort, and muscle pain. For computational reasons, the three outcomes, measured on the Borg scale, were rescaled such that they ranged between 0 and 1. The association with the outcomes was tested for the following pulmonary function parameters: VO_2_ peak (mL/min and mL/kg/min), WR (watts), heart rate [HR peak (bpm, %pred)], O_2_ pulse peak (mL/min/beat), HR/VO_2_ slope, VO_2_/work slope, minute ventilation [V_E_ peak (L)], breathing reserve as % of maximum voluntary ventilation [BR %MVV], V_E_/VCO_2_ slope, V_E_/VO_2_ slope, respiratory exchange ratio (RER max), P_a_CO_2_ at rest (mmHg), P_a_CO_2_ peak (mmHg), P(a–ET)CO_2_ peak (mmHg), A-aO_2_ peak (mmHg), P_a_O_2_ at rest (mmHg), P_a_O_2_ peak (mmHg), pH at rest and peak, bicarbonate [HCO_3_^−^] at rest and peak (mmol/L), base excess (BE) at rest and peak (mmHg), [K^+^] at rest and peak (mmol/L), BMI, age, gender. To understand how the pulmonary function parameters selected by the Boruta algorithm impact the symptoms on the Borg scale, we grew a CART for each outcome using the predictors identified by the algorithm. The CARTs were tuned using repeated k-fold cross-validation, setting the number of folds equal to 5 and repeating the operation 10 times [13].

Statistical analysis was performed using R statistical software (version 3.5.2) [14]. The Boruta algorithm was implemented using the *Boruta* R package (version 6.0.0) [15], whereas the CARTs were implemented using the *rpart* R package (version 4.1-10) [16].

## Results

Descriptive statistics of the patients’ anthropometric and clinical characteristics and PFTs data are shown in Tables 1 and 2, respectively. Out of 24 LTx recipients, 18 (75%) were men and 6 (25%) woman.

**Table 1:** Descriptive statistics of the patient’s characteristics

Variable	Value
Age, years	41 [30; 48]
BMI, kg/m^2^	23.14 [20.01; 24.82]
WR, W	93 [78; 103.5]
VO_2_ peak mL/min	1141 [1004; 1271]
VO_2_ mL/kg/min	15.6 [14.5; 21.3]
RER max	1.23 [1.17; 1.34]
HR peak, bpm	139 [129; 144]
O_2_ pulse peak, mL/beat	8.4 [7.6; 9.5]
VO_2_/W slope	8.8 [8.3; 9.2]
HR/VO_2_ slope	2.7 [21.5; 36.5]
VE/VCO_2_ slope	29.3 [27.2; 31.3]
VE/VO_2_ slope	34 [27.7; 38.8]
VE peak, L	48.4 [32.1; 60.4]
BR %MVV	46 [32; 53]
P(a-ET)CO_2_ peak, mmHg	-2.6 [-5.25; -0.85]
A-aO_2_ peak, mmHg	21.5 [16.5; 30.2]
pH rest	7.42 [7.41; 7.44]
pH peak	7.36 [7.33; 7.38]
PaCO_2_ rest, mmHg	34.6 [32.6; 35.6]
PaCO_2_ peak, mmHg	30.0 [26.4; 32.0]
PaO_2_ rest, mmHg	89.2 [86.3; 95.8]
PaO_2_ peak, mmHg	101.2 [89.5; 110.7]
HCO_3_^−^ rest, mmol/L	23.3 [21.7; 24.3]
HCO_3_^−^ peak, mmol/L	18.4 [16.7; 20.0]
BE rest, mmol/L	-2.2 [-3.2; -0.5]
BE peak, mmol/L	-8.3 [-10.3; -5.2]
K^+^ rest, mmol/L	4.22 [3.99; 4.54]
K^+^ peak, mmol/L	5.35 [5.02; 5.48]
Gender (male)	18 (0.75)
Gender (female)	6 (0.25)

Continuous variables are represented with median [I quartile, III quartile]. Categorical variables are represented with frequencies (percentages)

**Table 2:** PFTs distributions

Variable	Overall
n	24
VC (L) (median [IQR])	3.385 [3.100, 4.088]
VC (% pred) (median [IQR])	82.50 [76.50, 90.50]
FVC (L) (median [IQR])	3.325 [2.950, 4.036]
FVC (% pred) (median [IQR])	82.00 [75.75, 93.25]
FEV1 (L) (median [IQR])	2.81 [2.27, 3.44]
FEV1 (% pred) (median [IQR])	84.00 [69.75, 98.00]
FEV1/VC (%) (median [IQR])	84.42 [71.65, 89.54]
FEV1/VC (% pred) (median [IQR])	105.50 [89.75, 113.00]
FEV1/FVC (%) (median [IQR])	87.49 [75.473, 91.498]
TLC (L) (median [IQR])	5.020 [4.560, 6.203]
TLC (% pred) (median [IQR])	91.50 [77.00, 100.00]
RV/TLC (%) (median [IQR])	34.62 [29.79, 36.35]
RV/TLC (% pred) (median [IQR])	111.00 [93.75, 132.50]
DLCO (mL/min • mmHg) (median [IQR])	19.23 [16.94, 22.66]
DLCO (% pred) (median [IQR])	65.00 [60.00, 72.00]
KCO (mL/min • mmHg) (median [IQR])	3.74 [3.35, 4.68]
KCO (% pred) (median [IQR])	79.50 [70.25, 96.00]

Continuous variables are represented with median [I quartile, III quartile]. Categorical variables are represented with frequencies (percentages)

PFTs showed lung volumes close to the lower limit of normal values, in particular dynamic lung volumes. A slight reduction of diffusion capacity of carbon monoxide was documented. ABGs analysis showed normal P_a_O_2_ and P_a_CO_2_ values at rest and an increase of P_a_O_2_ and a reduction of P_a_CO_2_ at peak exercise. A mild compensated metabolic acidosis was present at rest and deteriorated at peak exercise; in parallel, arterial [K^+^] increased. Heart rate (HR) and HR/VO_2_ slope were consistent with an adequate chronotropic response to exercise. No sign of ventilation-perfusion mismatch was detected at peak exercise according to A-aO_2_ and P(a-ET)CO_2_. A slight degree of ventilatory inefficiency (V_E_/VCO_2_ slope) was found. An elevated respiratory exchange ratio (RER) was consistent with a maximal metabolic effort. A marked reduction in exercise capacity expressed in terms of WR and VO_2_ peak was found (around 40–60% of the predicted values).

**Fig. 1:**
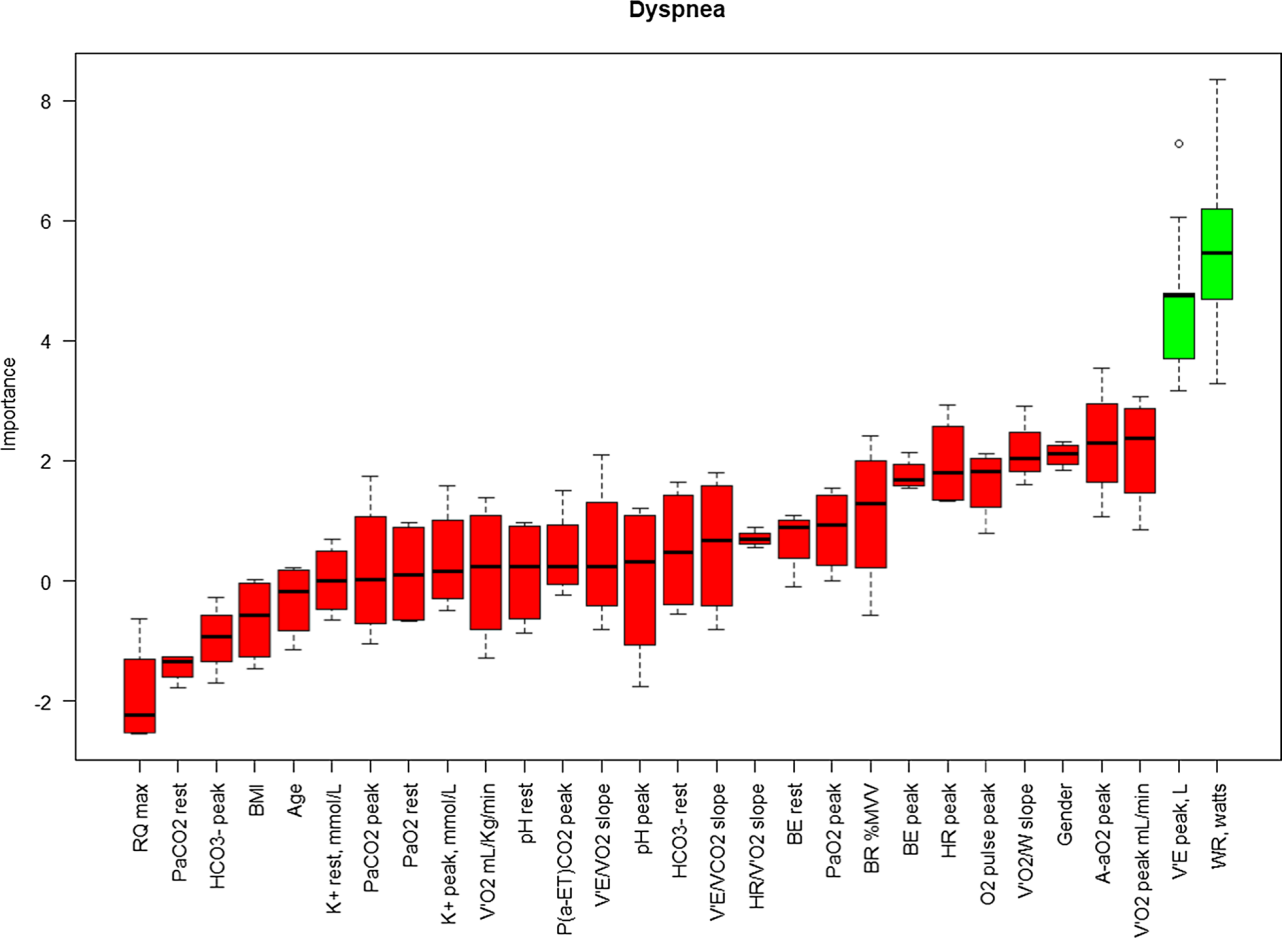
Variable importance Boruta dyspnea at the peak. On the x-axis the pulmonary function parameters are reported, whereas Z-score are reported on the y-axis. Boxplots represent the distribution of Z-score for each parameter returned by the algorithm. Green boxplots identify the parameters selected by the algorithm, whereas red boxplots denote the parameters discarded by the algorithm

**Fig. 2:**
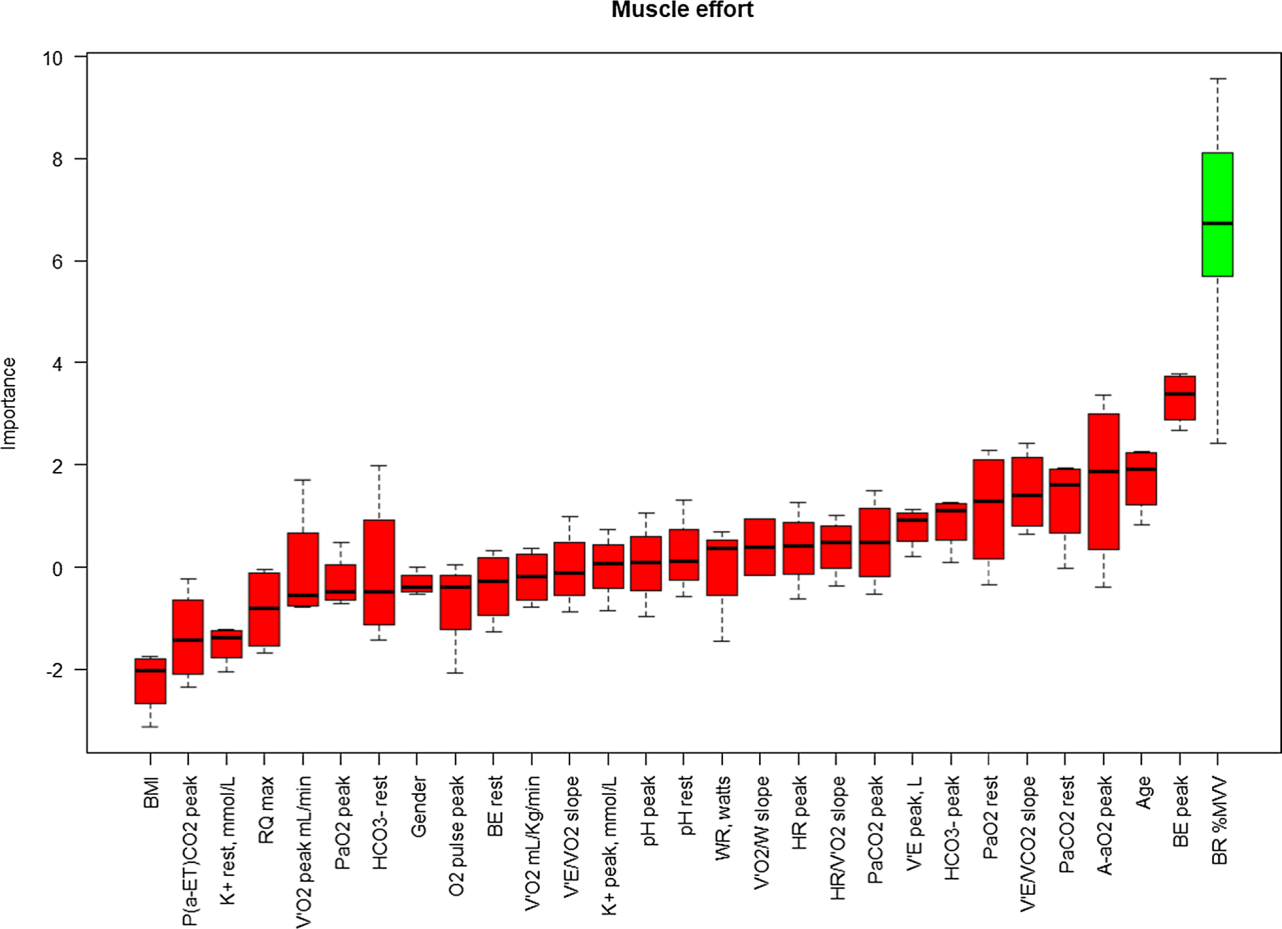
Variable importance Boruta for muscle effort at the peak. On the x-axis the pulmonary function parameters are reported, whereas Z-score are reported on the y-axis. Boxplots represent the distribution of Z-score for each parameter returned by the algorithm. Green boxplots identify the parameters selected by the algorithm, whereas red boxplots denote the parameters discarded by the algorithm

**Fig. 3:**
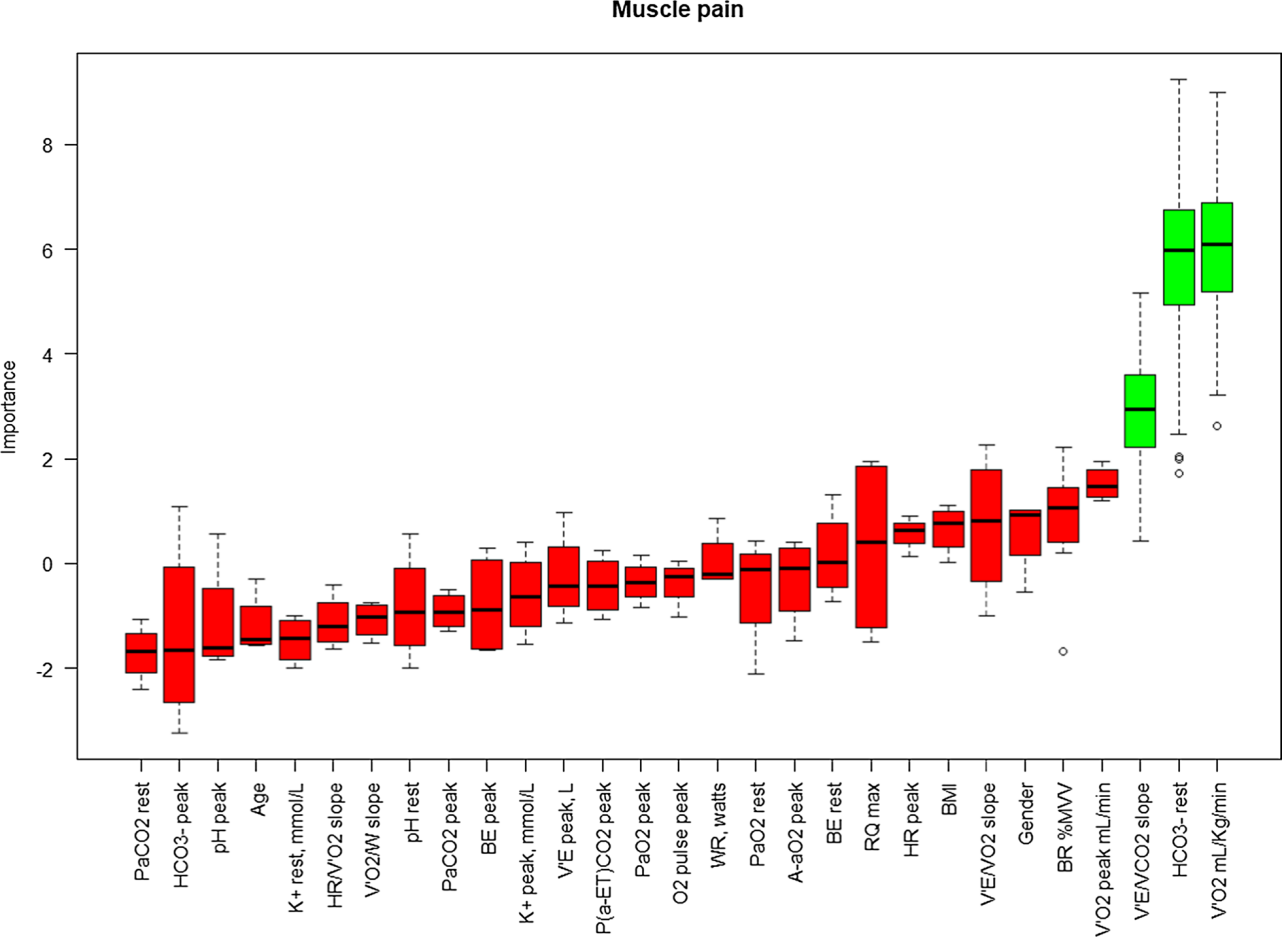
Variable importance Boruta for muscle pain at the peak. On the x-axis the pulmonary function parameters are reported, whereas Z-score are reported on the y-axis. Boxplots represent the distribution of Z-score for each parameter returned by the algorithm. Green boxplots identify the parameters selected by the algorithm, whereas red boxplots denote the parameters discarded by the algorithm

The results of the Boruta algorithm are shown in Figs. 1, 2, and 3 for dyspnea, muscle effort, and muscle pain, respectively. Cardiopulmonary function parameters selected by the algorithm are represented in green, whereas not selected parameters are shown in red. Dyspnea score was found to be significantly associated with both V_E_ (L/min) at peak exercise and maximum WR. Muscle effort score was significantly associated with BR%MVV, whereas muscle pain score was significantly associated with VO_2_ peak (mL/Kg/min), arterial [HCO_3_^−^] at rest, and V_E_/VCO_2_ slope.

**Fig. 4:**
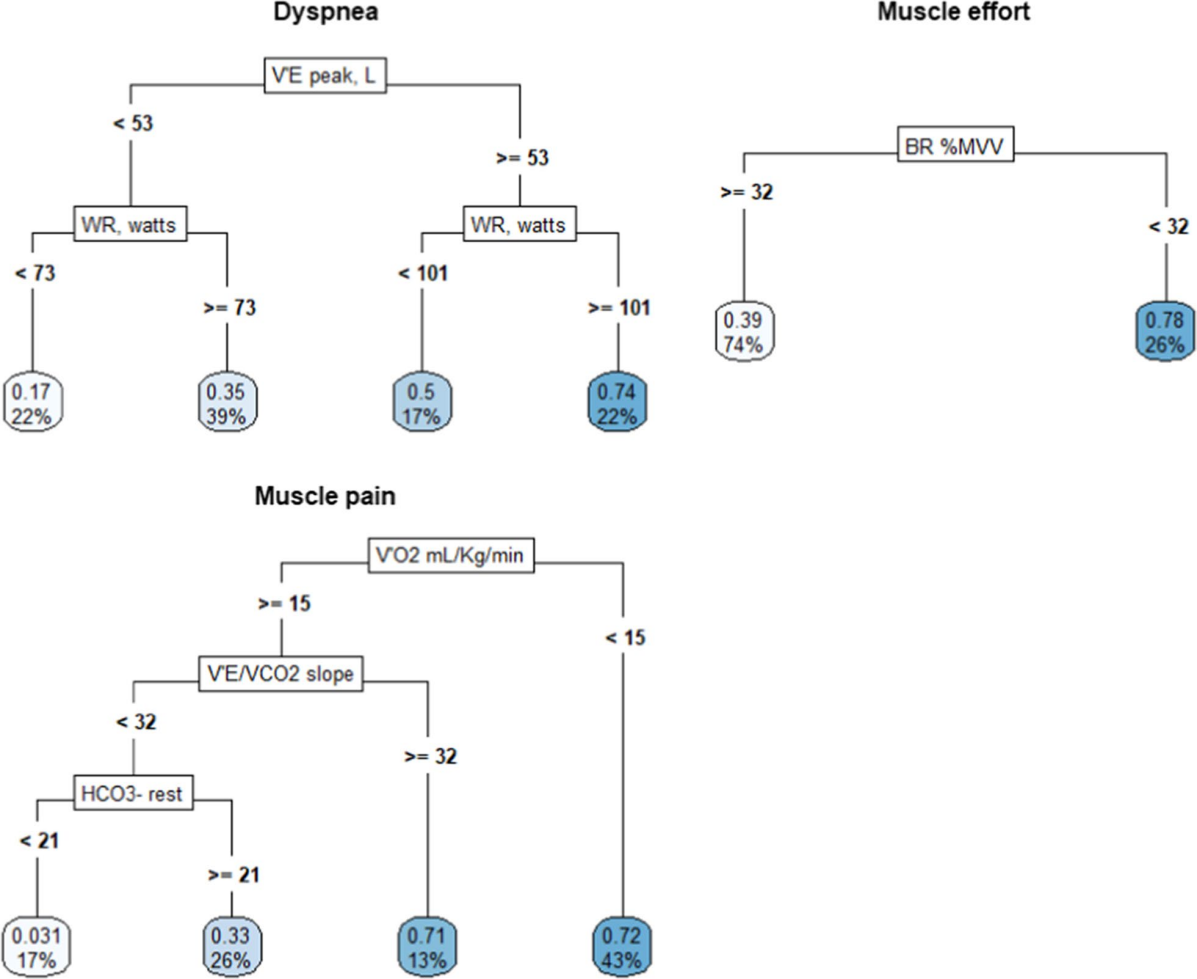
Graphical representation of CARTs grown for each outcome using the pulmonary function parameters identified by the Boruta algorithm. In the rectangular boxes the pulmonary function parameters used to split the tree are depicted. Values reported at both sides of the boxes represent the parameters values at which the split was performed. Bottom boxes are the “leaves” of the tree. They show the mean values of the Borg scale (ranging between 0 and 1) and the percentages of patients with that belong to that leaf

In Fig. 4 the CARTs grown for each outcome are represented. The CPET parameters used by the tree to perform each split are shown in the rectangular boxes. At both sides of the rectangular boxes, there are the “branches” of the trees, created by performing the split on the values of the parameters shown near the branches. The bottom boxes represent the “leaves” of the tree. They contain the mean outcome values on the Borg-scale (ranging between 0 and 1) and the percentages of patients assigned by the tree in that leaf, respectively. To interpret the impact of the parameters, trees must be read from top to bottom.

Regarding dyspnea, patients with V_E_ at peak lower than 53 L/min (61% of the patients) were further stratified by WR. LTx recipients with a value less than 73 watts (22%) showed an average Borg-scale value of 0.17 (2 on the original scale), whereas those with values equal or higher than 73 watts (39%) had an average Borg-scale value of 0.35 (between 3 and 4 on the original scale). Patients with V_E_ at peak equal or higher than 53 L/min were further divided into patients with WR values higher and lower than 101 watts. Those who showed values equal or higher than 101 watts (22%) showed an average Borg-scale value of 0.74 (between 7 and 8 on the original scale), whereas those that had values lower than 101 watts showed an average Borg-scale of 0.5 (between 5 and 6 on the original scale).

Regarding muscle effort, patients with BR%MVV equal or higher than 32 (74%) showed an average Borg-scale of 0.39 (4 on the original scale), whereas those with BR%MVV lower than 32 (26%) had higher Borg-scale values (8 on the original scale). Finally, regarding muscle pain, subjects with VO_2_ peak (mL/Kg/min) lower than 15 (43%) showed a Borg-scale value equal to 0.72 (7 on the original scale). Patients with VO_2_ peak (mL/Kg/min) higher than 15 and V_E_/VCO_2_ slope values equal or higher than 32 (13%) had a Borg-scale value equal to 0.71 (7 on the original scale). Subjects with VO_2_ peak (mL/Kg/min) equal or higher than 15, V_E_/VCO_2_ slope values lower than 32 and arterial [HCO_3_^−^] at rest lower than 21 (17%) had an outcome value of 0.031 (nearly 0 on the original scale), whereas individuals with VO_2_ peak (mL/Kg/min) equal or higher than 15, V_E_/VCO_2_ slope values lower than 32 and arterial [HCO_3_^−^] at rest higher than 21 (26%) had an outcome value of 0.33 (3 on the original scale).

## Discussion

We investigated the course of symptoms associated to exercise limitation and their relationships with CPET parameters during an incremental exercise testing in a group of uncomplicated LTx recipients.

In our series, we found a reduction of exercise performance in terms of maximum WR and VO_2_ peak (mL/Kg/min) compared to the reference values which was related to peripheral muscle limitation, similarly to previous results [1–3]. Additionally, we analyzed symptoms perception at rest and peak exercise by the modified Borg Scale, identifying a significant association with a number of CPET parameters. Because muscle pain at peak exercise was a commonly reported limiting symptom by some of the LTx recipients, we separately evaluated muscle pain from muscle effort in addition to breathing effort perception (dyspnea) (Figs. 1, 2, and 3).

### Factors associated to exercise limitation in LTx recipients according to current literature

Following LTx, despite the improvement of the ventilatory limitation, peak exercise capacity remains substantially lower than predicted values, and skeletal muscle dysfunction has been implicated as the primary factor limiting exercise in this type of patients [17]. A decreased proportion of oxidative (type 1) muscle fibers of the quadriceps muscles, a lower concentration of oxidative enzymes, an impaired oxygen extraction, an earlier drop in muscle pH with exercise, and a reduced muscle strength were demonstrated in several studies [18–20]. A physiological study performed by Pantoja and coworkers suggested that weakness of the lower limbs played a role on exercise intolerance in a group of stable LTx recipients [2]. In a clinical study, Kugler and coworkers found that muscle weakness perception was one of the most frequent symptoms reported by LTx recipients, strictly related to their QoL [3]. In a review on the use of CPET in LTx recipients, Dudley and El-Chemaly reported that lower limb fatigue was overwhelmingly the predominant reason for termination of exercise, suggesting that the primary exercise limitation was located at the level of oxygen extraction [21]. Also, they suggested that many factors such as underlying disease, drug treatments (e.g. chronic use of corticosteroids and cyclosporine), pretransplant chronic muscle dysfunction and rehabilitation programs before LTx, could affect peak VO_2_ and WR after transplantation. Finally, in group of LTx recipients, Spiesshoefer and coworkers recently observed that respiratory muscle function, especially diaphragm, was impaired, linked to elevated TNF-α levels and associated with exercise intolerance [22].

### Symptom perception and exercise limitation in our LTx recipients

Regarding muscle pain, LTx recipients with a poorer exercise performance in terms of VO_2_ peak (lower than 15 mL/kg/min, 43% of the subjects) reported a higher symptoms perception at peak exercise as well as those able to undergo a better exercise performance (more than 15 mL/kg/min) but requiring a higher cost of ventilation to get on with exercise. Conversely, the perception of muscle pain was very mild at the Borg scale in the group of LTx recipients with a better performance in terms of VO_2_ peak (> 15 mL/kg/min) and a lower energy cost of ventilation (V_E_/VCO_2_ < 32), independently of the level of metabolic acidosis at rest (arterial [HCO_3_^−^]). Therefore, the LTx recipients most prone to have muscle pain at peak exercise were those with poorer aerobic capacity or those who required a higher energy cost of ventilation to compensate exercise-related metabolic acidosis.

BR%MVV was the only functional parameter significantly associated to muscle effort. LTx recipients with a lower BR%MVV at peak exercise reported more muscle effort perception compared to those who had a higher breathing reserve. The BR%MVV is a component of the overall pulmonary adaptation to exercise, strictly related to V_E_. As for the chronic airway obstructive syndromes, a pre-existent reduction in lung function, an altered breathing pattern and a faster increase in ventilatory demands during exercise can lead to an earlier exhaustion of breathing reserve and consequently a reduced ventilatory support to meet the metabolic needs related to muscle effort. As a result, muscle effort appears as intolerable limiting symptom during exercise. We did not find any other significantly associated CPET parameter to muscle effort perception. Furthermore, we also report that respiratory muscle function measurements were not assessed, representing a limitation of this study.

Dyspnea perception was driven by the peak V_E_ and WR. A higher ventilation in response to higher WR was associated to a progressive increase of dyspnea perception, as expected [23, 24].

In LTx recipients who reached lower V_E_ peak values (less than 53 L/min) and lower WR, dyspnea was not a limiting symptom (Borg scale between 2 and 4, from “slight” to “somewhat severe”), suggesting a prevalent role of peripheral limitation to maximal exercise. LTx recipients who reached higher level of WR (≥ 101 W) where also those who showed higher levels of V_E_ (≥ 53 L/min) and this pattern was associated to the highest perception of dyspnea (between 7 and 8 at the Borg scale, “very severe” or more), suggesting that this was the limiting symptom in this subgroup of patients (Figs. 1 and 4).

### Lung function, blood gases and acid–base balance in our LTx recipients

PFTs at rest were overall within the normal range, suggesting a preserved graft function (Table 2). The absence of a significant ventilatory limitation or ventilation-perfusion mismatch during exercise (Table 1) was consistent with stable clinical condition. ABGs at rest were consistent with a mild compensated metabolic acidosis. In fact, the resting ABGs showed a reduction of arterial [HCO_3_^−^] and P_a_CO_2_ while pH was slightly reduced or normal. In our series, the presence of a mild chronic metabolic acidosis may depend on several factors, including pre-transplant condition, drugs interactions, post LTx complications [18, 19]. As a consequence, to restore normal arterial blood gases and central chemoreceptor homeostasis, ventilation is stimulated by pH until P_a_CO_2_ is reduced to a lower P_a_CO_2_ set-point [25, 26]. All these factors can affect the overall response to exercise in LTx recipients.

The metabolic response to incremental exercise in our LTx recipients showed an early appearance of lactate threshold and a faster increase in RER, reaching values consistent with a maximal exercise intensity and an expression of the anaerobic muscle metabolism involvement (very high peak RER value, average value of 1.23). In normal subjects a severe metabolic acidosis is induced by heavy exercise. Therefore, in the presence of metabolic acidosis at rest, the higher the WR, the higher the additional ventilation required for the task and an anticipation of the limiting symptoms occurs. In our LTx recipients, we found that arterial [HCO_3_^−^] at rest was significantly associated with muscle pain score suggesting that chronic metabolic acidosis at rest, altering the composition of muscle extracellular fluid, could be involved in the onset of muscle pain during exercise. Another physiological response to incremental exercise is a substantial increase in plasma [K^+^], released by contracting muscle, that has also been linked to the onset of muscle pain [26–28]. In our series we did not find a significant association between the arterial [K^+^] and symptoms perception, with specific reference to muscle pain. Differences between venous and arterial [K^+^] and/or in the distribution of [K^+^] among the storage systems may explain these finding.

### Methodological aspects: small sample size and high number of pulmonary function parameters

Small size studies can be very valuable in clinical research, especially in situations where ethical and practical concerns may arise and when a new research hypothesis is tested for the first time [29]. Nevertheless, the small number of observations in a study may represent a problem that must be faced in order to reliably answer the addressed questions.

In small sample size settings, classical statistical approaches, such as standard hypothesis tests and regression models, are known to have several limitations. Their underlying assumptions are often difficult to test in such situations and they are likely to be unreliable in clinical studies with few patients. As a matter of fact, the use of such tools can produce misleading and unstable results that can hardly help the development of effective clinical therapies for the population of analyzed individuals.

The small sample size issue becomes even harder to face when the number of subjects is lower than their considered characteristics (as in our study), whose association with a clinical endpoint is tested. In the statistical literature, this is known as the “n < p” problem, which represents a serious issue when standard statistical approaches are used to evaluate the impact of a large number of predictors [30]. Indeed, in these settings, the goal is to select a minimum set of explanatory variables, which are the most important in predicting the outcome of interest.

MLTs are increasingly being used in clinical research [31, 32]. Their modeling flexibility makes them valuable tools, especially to describe complex relationships between the outcome and the predictors. Furthermore, in contrast to the standard statistical methods, they do not make any parametric assumptions, which may be potentially advantageous in small studies where the assumptions of classical methods often do not hold.

The approach used in the paper showed that useful information could been extracted even from complex datasets, where complexity stems out from the combination of the richness of information and pauperism of patients. Clearly, when number of patients is limited, caution should be taken when interpreting data, e.g.: the statistical analysis by Boruta algorithm, which stratifies the functional parameters in order of importance, does not exclude a possible role of those parameters with a lower level of importance.

## Conclusions

Our study is the first, to our knowledge, that evaluated the possible association between the onset of symptoms and the course of CPET parameters in a group of uncomplicated LTx recipients applying the model of the MLTs. The majority of our LTx recipients reported peripheral limitation as the prevalent reason for exercise termination. Muscle pain at peak exercise was strictly associated with basal and exercise-induced metabolic altered pathways. The onset of dyspnea (breathing effort) was associated with the intensity of ventilatory response to meet metabolic demands for increasing WR. In conclusion, symptoms experienced during exercise in LTx recipients represent the combination of several mechanisms depending on a complex interaction between pre-transplant and post-transplant conditions, the comprehension of which requires further clarification.

Our study suggests that only an accurate assessment of symptoms combined with cardio-pulmonary parameters allows a correct interpretation of exercise limitation and a tailored exercise prescription. The role and mechanisms of muscle pain during exercise in LTx recipients requires further investigations.

## Data Availability

The datasets generated and/or analysed during the current study are not publicly available due to privacy law reasons but are available from the corresponding author on reasonable request.

## Abbreviations

A-aO_2_: Alveolar arterial partial pressure of oxygen difference
ABGs: Arterial blood gases
BE: Base excess
BR %MVV: Breathing reserve as % of maximum voluntary ventilation
CART: Regression and Classification Trees
CPET: Cardio-pulmonary exercise testing
DLCO: Diffusing capacity for carbon monoxide
FEV1: Forced expiratory volume in 1 second
FVC: Forced vital capacity
[K^+^]: Arterial potassium concentration
KCO: Carbon monoxide transfer coefficient
[HCO_3_^−^]: Arterial bicarbonate concentration
HR: Heart rate
LTx: Lung transplant
MLTs: Machine learning techniques
P_a_CO_2_: Partial pressure of carbon dioxide
P(a–ET)CO_2_: Arterial end-tidal partial pressure of carbon dioxide difference
P_a_O_2_: Partial pressure of oxygen
PFTs: Pulmonary function testing
RER: Respiratory exchange ratio
RF: Random forest
RV: Residual volume
TLC: Total lung capacity
VC: Vital capacity
VCO_2_: Carbon dioxide production
V_E_: Minute ventilation
VO_2_: Oxygen uptake
WR: Maximum power output
6MWT: Six-minute walking test
